# Optimization of Multimodal Imaging of Mesenchymal Stem Cells Using the Human Sodium Iodide Symporter for PET and Cerenkov Luminescence Imaging

**DOI:** 10.1371/journal.pone.0094833

**Published:** 2014-04-18

**Authors:** Esther Wolfs, Bryan Holvoet, Rik Gijsbers, Cindy Casteels, Scott J. Roberts, Tom Struys, Michael Maris, Abdelilah Ibrahimi, Zeger Debyser, Koen Van Laere, Catherine M. Verfaillie, Christophe M. Deroose

**Affiliations:** 1 Nuclear Medicine and Molecular Imaging, Department of Imaging and Pathology, KU Leuven, Leuven, Belgium; 2 Laboratory of Molecular Virology and Gene Therapy, Department of Pharmaceutical and Pharmacological Sciences, KU Leuven, Leuven, Belgium, and Leuven Viral Vector Core, KU Leuven, Leuven, Belgium; 3 Skeletal Biology and Engineering Research Center, Department of development and regeneration, KU Leuven, Leuven, Belgium; 4 Lab of Histology, Department of morphology, Biomedical Research Institute, Universiteit Hasselt, Hasselt, Belgium; 5 Stem Cell Institute Leuven, Department of development and regeneration, KU Leuven, Leuven, Belgium; Rutgers - New Jersey Medical School, United States of America

## Abstract

**Purpose:**

The use of stably integrated reporter gene imaging provides a manner to monitor the *in vivo* fate of engrafted cells over time in a non-invasive manner. Here, we optimized multimodal imaging (small-animal PET, Cerenkov luminescence imaging (CLI) and bioluminescence imaging (BLI)) of mesenchymal stem cells (MSCs), by means of the human sodium iodide symporter (hNIS) and firefly luciferase (Fluc) as reporters.

**Methods:**

First, two multicistronic lentiviral vectors (LV) were generated for multimodal imaging: BLI, ^124^I PET/SPECT and CLI. Expression of the imaging reporter genes was validated *in vitro* using ^99m^TcO_4_^−^ radioligand uptake experiments and BLI. Uptake kinetics, specificity and tracer elution were determined as well as the effect of the transduction process on the cell's differentiation capacity. MSCs expressing the LV were injected intravenously or subcutaneously and imaged using small-animal PET, CLI and BLI.

**Results:**

The expression of both imaging reporter genes was functional and specific. An elution of ^99m^TcO_4_^−^ from the cells was observed, with 31% retention after 3 h. After labeling cells with ^124^I *in vitro*, a significantly higher CLI signal was noted in hNIS expressing murine MSCs. Furthermore, it was possible to visualize cells injected intravenously using BLI or subcutaneously in mice, using ^124^I small-animal PET, CLI and BLI.

**Conclusions:**

This study identifies hNIS as a suitable reporter gene for molecular imaging with PET and CLI, as confirmed with BLI through the expression of Fluc. It supports the potential for a wider application of hNIS reporter gene imaging and future clinical applications.

## Introduction

The development of imaging reporter genes is of great importance to the field of molecular imaging, as their encoded proteins can permit longitudinal non-invasive imaging of biological processes at the cellular and subcellular level.

One of these imaging reporter genes encodes for the well-characterized human sodium iodide symporter (hNIS) [1]. hNIS is situated in the basolateral membrane of thyroid follicular cells, as well as other tissues such as the stomach mucosa, salivary glands and the lactating mammary gland. hNIS is an intrinsic plasma membrane glycoprotein with 13 transmembrane domains that mediates the first step in the formation of thyroid hormones by the transport of iodide into the thyroid follicular cells against a concentration gradient [1]. Furthermore, hNIS is able to transport radioactive forms of iodide, as well as other anions such as technetium pertechnetate (^99m^TcO_4_^−^) [2]. Due to its relatively low endogenous expression in extrathyroidal tissues, the wide availability of FDA-approved radioactive probes and the experience with hNIS imaging, hNIS satisfies most of the criteria to be a suitable reporter gene for imaging purposes [3]. Furthermore, hNIS is a human protein and its use as an imaging reporter gene will therefore not evoke immunological responses [4].

Exogenous expression of hNIS can be applied for non-invasive and nuclear imaging of grafted cells. This can lead to a better understanding of stem cell homing, and assist in further applying and developing stem cell-based therapies.

Mesenchymal stem cells (MSCs) are non-hematopoietic multipotent stem cells with an innate ability to differentiate towards multiple mesenchymal lineages [5], [6]. These cells appear to be good candidates for clinical use; they can be expanded easily *in vitro* and lack immunogenicity [7]. MSCs also possess immune modulating properties, through the inhibition of immune cell function and proliferation, and their use as immunomodulators is being explored clinically [8]. Besides their role in tissue regeneration, MSCs have significant trophic effects on endogenous (stem) cells [9]. Furthermore, they have also been proven to migrate towards multiple tumors *in vivo*, which renders them good candidates as delivery vehicles for antitumor therapy [10]–[12].

Because of these characteristics, the interest in MSCs for clinical applications has increased over the past years. As such, MSCs have been used in several clinical phase I and phase II studies concerning acute myocardial infarction [13], but the most promising results have been obtained in human graft-versus-host disease and allograft rejection studies [14]–[18].

To further improve stem cell-based treatments, cellular homing as well as survival after engraftment needs to be studied in greater detail. Imaging reporter genes can play an important role in the non-invasive longitudinal follow-up of grafted cells. In the present work, we optimized lentiviral vector (LV) transduction of murine MSCs, inducing the expression of the imaging reporter genes firefly luciferase (Fluc) for bioluminescence imaging and hNIS for emission tomography (PET/SPECT) and Cerenkov luminescence imaging (CLI).

CLI uses Cerenkov radiation for molecular imaging. Cerenkov radiation is an electromagnetic radiation emitted when a charged particle travels at a speed beyond the speed of light in a dielectric medium. The upper limit on speed (c), is the speed of light in a vacuum, but in a particular medium the speed of light is a fraction of c (in water ∼0.75), and positrons from PET isotopes can be emitted at speeds higher than these values. The charged particle travels through the medium and thereby temporarily displaces the electrons in the medium. While returning to their ground state, the electrons will emit visible light photons that can be detected using a BLI system [19], [20]. It is thus possible to image certain PET radionuclides via an optical system with high sensitivity and short scanning times.

Here, the longitudinal expression of these imaging reporter genes was evaluated both *in vitro* and *in vivo*.

## Materials and Methods

### Cell culture

HEK293T cells were used for testing the functionality of the different vectors described below. Cells were cultured under normal oxygen conditions in a 5% CO_2_ humidified incubator at 37°C. Growth medium consisted of Dulbecco's modified eagle medium (DMEM, Gibco, Invitrogen, Carlsbad, CA, USA), 10% fetal bovine serum (FBS; Lonza BioWhittaker, Basel, Switzerland) and 1% Penicillin/Streptomycin (Gibco).

Murine MSCs from C57Bl/6 mice were obtained from the lab of Prof. dr. D Prockop, Tulane University, USA [21]. Cells were cultured in a 5% CO_2_ humidified incubator under normoxic conditions at 37°C in growth medium containing Iscove's Modified Dulbecco's Medium (IMDM, Gibco), 10% FBS, 10% horse serum (Biochrom, Berlin, Germany), 1% L-glutamine (Gibco) and 1% penicillin/streptomycin.

### Lentiviral vector optimization and transduction

**Figure 1:**
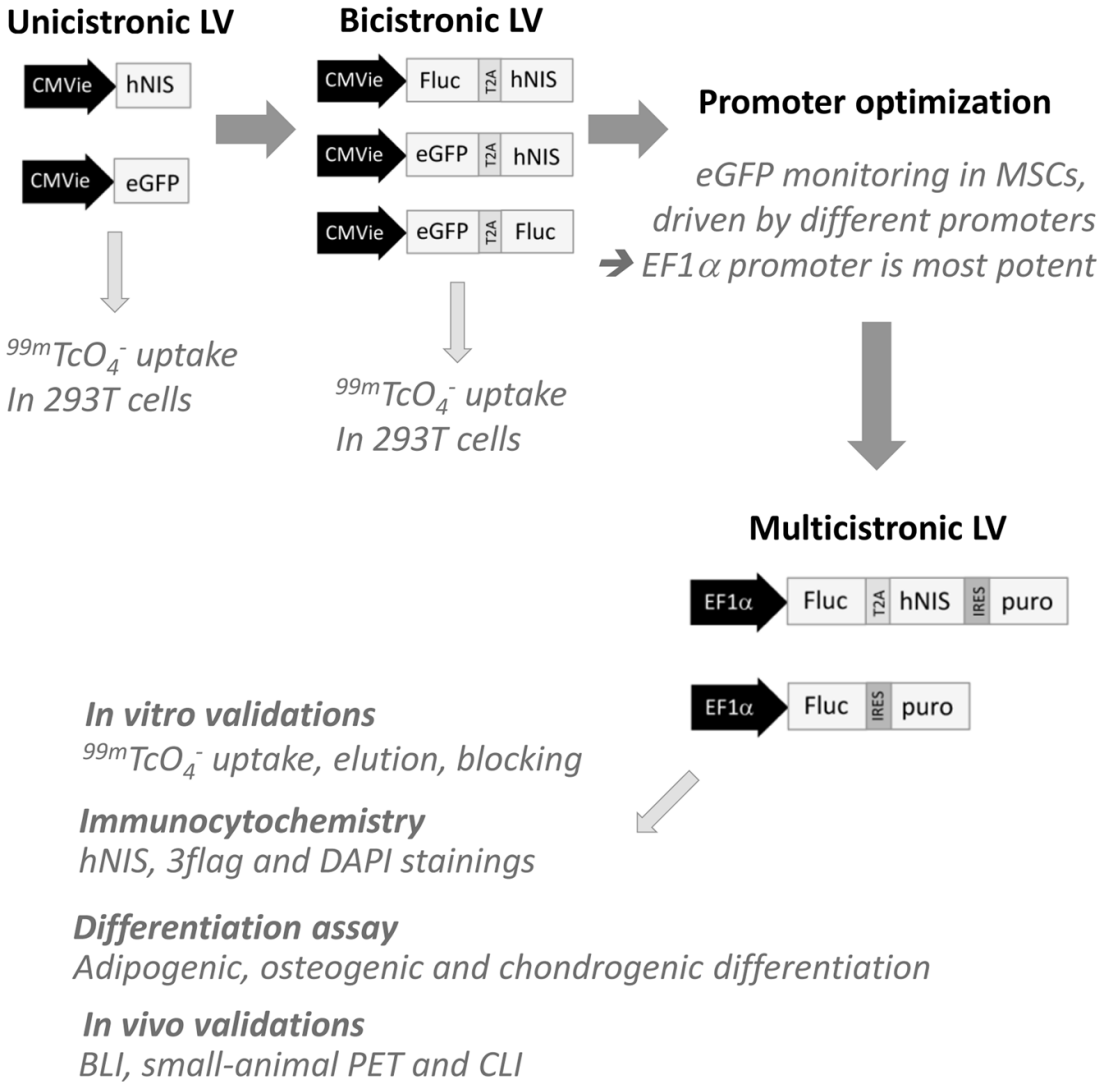
Diagram illustrating the steps involved in this study. Overview of the lentiviral vector constructs used to induce reporter gene expression and the experiments performed with these respective vectors.

For the optimization of the final multicistronic LV construct, different vector types and constructs were generated and tested, (for an overview see Table 1 and Fig. 1). LV constructs carrying a cytomegalovirus immediate early (CMVie) promoter driving hNIS or enhanced green fluorescent protein (eGFP) as a control (LV_hNIS and LV_eGFP, respectively) were used at first in HEK293T cells as a proof-of-principle to demonstrate hNIS functionality. Subsequently, bicistronic LV were generated, combining hNIS with alternative imaging reporter modules where a CMVie promoter drives hNIS and Firefly luciferase (Fluc, for bioluminescence imaging) or hNIS and eGFP (fluorescence) coupled by a *Thosea asigna* virus 2A (T2A) sequence, LV_Fluc-T2A-hNIS and LV_eGFP-T2A-hNIS, respectively. As a control, a LV was included encoding both eGFP and Fluc coupled by a T2A sequence, LV_eGFP-T2A-Fluc [22]. In parallel, we determined the ideal promoter for efficient MSC transcription, employing a LV that drives eGFP from different promoters, such as human elongation factor 1α (EF1α), human Cyclophilin A (CypA), or viral Spleen focus forming virus LTR (SFFV), and CMVie. These LV were referred to as LV_hEF1α-eGFP, LV_CypA-eGFP, LV_SFFV-eGFP, LV_CMVie-eGFP, respectively. MSCs were transduced with the respective vectors using a protocol as reported earlier [23]. eGFP fluorescence was monitored using fluorescence activated sorting (FACS) and on day 37, the 5% brightest population was isolated from each condition to overcome the lack of puromycin resistance in the expression cassettes. Long-term eGFP expression was monitored using FACS until 60 days. Results are given as total fluorescence (fraction of total cells that are fluorescent×mean fluorescence intensity).

**Table 1:** Vector constructs used for gene transfer.

Vector type	Expression cassette	Cell type
LV	LV_hNIS	HEK293T
	LV_eGFP	
Bicistronic LV	LV_hNIS-T2A-Fluc	HEK293T
	LV_hNIS-T2A-eGFP	
	LV_Fluc-T2A-eGFP	
Multicistronic LV	LV_hEF1α-3flagFluc-T2A-hNIS-IRES-PuroR	MSCs
	LV_EF1α-3flagFluc-IRES-PuroR	

Finally, a multicistronic LV carrying the hEF1α promoter to drive triple flag tagged firefly luciferase (3flagFluc), a T2A sequence, the human sodium iodide symporter (hNIS), an internal ribosomal entry site (IRES), and a puromycin resistance gene (PuroR), LV_hEF1α-3flagFluc-T2A-hNIS-IRES-PuroR was tested. As a control, the same vector lacking hNIS was used, LV_EF1α-3flagFluc-IRES-PuroR. For the sake of clarity, MSCs transduced with the LV_EF1α-3flagFluc-T2A-hNIS-IRES-PuroR will be referred to as Fluc-hNIS expressing MSCs. MSCs transduced with the EF1α-3flagFluc-IRES-Puro LV, will be referred to as Fluc expressing MSCs.

To create stable cell lines, cells were seeded into medium containing serial dilutions of LV and cells were incubated for 48 hours. For the selection of transduced cells, 2 µg/mL puromycin (Merck Millipore, Darmstadt, Germany) was added to the growth medium, and cells were maintained under this condition.

### *In vitro* radiotracer uptake experiments

Cells were plated in triplicate in 24-well plates at a density of 10^5^ cells per well in normal growth medium, and kept under standard incubation conditions. After 24 hours, cells were washed with PBS and incubated with 250 µL of pertechnetate (^99m^TcO_4_^−^) tracer solution (0.74 MBq/mL in DMEM; Gibco) for different periods (n = 3). All *in vitro* data are shown as decay-corrected values.

After incubation, cells were washed 3 times with phosphate buffered saline (PBS; Gibco), and tracer concentration in the cell fraction was measured using a gamma counter (Perkin Elmer, Waltham, MA, USA). Uptake values were corrected for the cell number in the according samples, as measured using a nucleocounter system (Chemometec, Allerød, Denmark).

The elution of ^99m^TcO_4_^−^ initially taken up by the cells was measured by incubating the cells for one hour with ^99m^TcO_4_^−^ (0.74 MBq/mL), washing the cells and incubating them on tracer-free DMEM for varying periods. The activity in the cells, the supernatant and the elution medium was measured and elution rates were calculated (n = 3).

A blocking experiment was performed with sodium perchlorate (NaClO_4_) using three different concentrations solved in DMEM: 10, 25 and 50 µM. The cells were incubated with the blocking solutions containing the tracer (0.74 MBq/mL ^99m^TcO_4_^−^ in DMEM+ NaClO_4_) and activity in supernatant and cells was measured using a gamma counter (n = 3).

### Immunocytochemistry

To further confirm hNIS and 3flag expression in MSCs transduced with the LV_EF1a-3flagFluc-T2A-hNIS-IRES-PuroR and the LV_EF1a-3flagFluc-IRES-PuroR, immunofluorescent stainings were performed. Cells were fixed using unifix for 20 min at 4°C, permeabilized with 0.05% Triton for 30 min at room temperature (only in case of 3flag staining) and blocked with 10% normal donkey serum (Millipore) for 20 min at room temperature. Cells were then incubated for two hours at room temperature with the primary antibody diluted in PBS (hNIS 1/20 and flag 1/1000). Fluorescently labeled secondary antibody (1∶500, Alexa Fluor donkey anti-rabbit 488 or donkey anti-mouse 555, Invitrogen) was incubated for 30 min at room temperature. Nuclei were counterstained using DAPI and sections were mounted using anti-fade mounting medium (Dako). Images were acquired using a Nikon Eclipse 80 i Fluorescence microscope equipped with a Nikon DS-2 MB Wc digital camera (Nikon Tokyo, Japan). Stainings in which primary antibodies were omitted, were used as a negative control.

### MSC differentiations

For adipogenic differentiations, cells were plated at a density of 10400 cells/cm^2^ in differentiation medium, containing αMEM (Gibco), 10% FBS, 100 units of penicillin and 1000 units of streptomycin, 1 µM dexamethasone, 10 µg/mL human insulin, 100 µM indomethacin and 25 µM methyl-isobutylxanthine (all from Sigma-Aldrich, St Louis, MO, USA). Medium was changed twice a week for 14 days (n = 3).

After 14 days, cells were rinsed with PBS, fixed using unifix (Klinipath, Duiven, The Netherlands) for 20 minutes and stained with fresh Oil red O solution (Sigma-Aldrich) for 10 minutes. Images were taken using an inverted Zeiss axiovert microscope (Hertfordshire, UK). For quantification of the lipid droplet staining, the dye was extracted from the cells using 100% ethanol and aliquots of 200 µL were transferred to a 96-well plate in triplicate. Absorbance was measured at 450 nm using a Victor 1420 plate reader (Perkin Elmer).

To assess the effect on osteogenic differentiation capacity, MSCs were seeded at 8400 cells/cm^2^ in normal growth medium and allowed to become confluent for 48 h before adding osteogenic differentiation medium. Differentiation medium contained DMEM with sodium pyruvate, 10% FBS, 1% penicillin/streptomycin, 50 µg/mL L-Ascorbic acid 2-phosphate sequimagnesium salt hydrate (AA-P; Sigma-Aldrich), 100 nM dexamethasone and 10 mM glycerol-2-phosphate disodium salt hydrate (bGP). Medium was changed twice a week for 3 weeks (n = 3).

At day 21, cells were rinsed with PBS, fixed with ice-cold 70% ethanol for 1 h and stained with Alizarin red S solution (Sigma) for 30 minutes. Images were taken using an inverted Zeiss axiovert microscope. Afterwards, the dye was extracted from the cells using a 10% cetylpiridinium chloride solution for 1 h. Aliquots of 200 µL were transferred to a 96-well plate in triplicate, and absorbance was measured at 560 nm.

For chondrogenic differentiation, micromasses were generated by seeding 20 µL droplets of cell suspension, each containing 200,000 cells, into separate wells of a 24-well plate. After attachment for 3 hours expansion medium was added. The next day, chondrogenic differentiation medium was added containing DMEM/F12, 2.5% FBS, 100 nM dexamethasone, 1× ITS+, 50 µg/mL AA-P, 10 ng/mL Transforming Growth Factor-β (TGF-β), 10 µM ROCK inhibitor and 40 µg/mL L-proline. Every 2 days, medium was changed for 21 days (n = 3).

At day 21, cells were fixed using unifix and stained overnight with Alcian Blue. The dye was extracted using 6M guanidine HCL. The optical density of the extracted dye was measured at 595 nm in triplicates.

### Animal preparations

The protocols used in this study were approved by the Ethical Committee of the KU Leuven (Permit Number: P145-2010).

For the intravenous injections, animals were anesthetized with 2% isoflurane (Isoflurane ISP®, Rothacher, Basel, Switzerland) in 100% oxygen, at a flow rate of 2 L/minute. Different cell numbers of MSCs expressing Fluc-hNIS ranging from 10,000 to 1,000,000 were injected in the tail vein of healthy C57BL/6 mice, and 30 minutes after cell injections BLI was performed (n = 3).

For xenografts, both Fluc expressing MSCs (as a control), and Fluc-hNIS expressing MSCs were injected subcutaneously in anesthetized nude athymic mice (nu/nu) (n = 3). Fluc expressing MSCs were injected on the left side of the body, whereas Fluc-hNIS expressing cells were injected on the right side of the body. Each mouse was injected with two different cell numbers per cell line: 10,000 and 1,000,000 mixed with matrigel in a 1∶1 ratio (BD biosciences, New Jersey, USA).

### Small-animal PET

The imaging of xenografts was performed on day 2 after xenograft generation with 11 MBq of ^124^I (Perkin Elmer) per mouse injected intravenously, with dynamic acquisitions of 90 minutes (n = 3) using a Focus 220 small-animal PET system (Siemens Medical Solutions USA, Knoxville,TN). Images were reconstructed with a maximum a posteriori (MAP) image reconstruction algorithm, and analyzed in PMOD 3.0 (PMOD technologies, Zürich, Switzerland). Images were converted to standardized uptake value (SUV) according to the standard formula: SUV = Activity concentration in organ/[injected activity/animal weight].

A manually delineated volume of interest (VOI) was positioned on the dynamic images over the Fluc-hNIS-expressing xenografts, the control Fluc-expressing xenografts, and muscle tissue and the brain as background tissues to generate time activity curves. Ratios were calculated, comparing the signal in the Fluc-hNIS expressing xenograft with background signals from control Fluc-expressing xenografts, muscle tissue and brain.

### Cerenkov luminescence imaging

For the *in vitro* CLI, cells were plated in triplicate in 24-well plates at a density of 1×10^5^ cells per well in normal growth medium, and kept under standard incubation conditions. After 24 hours, cells were washed with PBS and incubated with 250 µL of tracer solution (0.74 MBq/mL ^124^I in DMEM; Gibco) for one hour. Cells were washed 3 times with PBS, and placed in the BLI chamber for the acquisition of 1 minute scans.

The *in vivo* CLI scans on xenografts were performed daily after performing the ^124^I small-animal PET scans. CLI protocols were executed as follows: animals were anesthetized using isoflurane in 100% oxygen, at a flow rate of 2 L/minute and positioned in the BLI chamber without prior injection of D-luciferin. Images were acquired using an IVIS 100 system (Perkin Elmer) and CLI acquisition was done by acquiring one minute frames. The data are reported as total photon flux (p/s) from a circular region of interest (ROI). For all animals, first a daily CLI scan was performed to measure the ^124^I distribution, and thereafter D-luciferin was injected to measure the BLI signal intensity.

### Bioluminescence imaging

For the *in vitro* BLI, cells were plated in triplicate in 24-well plates at a density of 1×10^5^ cells per well in normal growth medium, and kept under standard incubation conditions. After 24 hours, cells were washed with PBS and incubated with 250 µL of D-luciferin (0.3 µg/mL; Promega, Benelux, Leiden, The Netherlands). Cells were placed in the BLI chamber immediately for the acquisition of 1 minute scans.

Animals were anesthetized with 2% isoflurane in 100% oxygen, at a flow rate of 2 L/minute, after which D-luciferin, dissolved in PBS (15 mg/mL), was injected intravenously (126 mg/kg body weight). Images were acquired using an IVIS 100 system (Perkin Elmer). Consecutive 1 minute frames were acquired until the maximum signal intensity was reached. Each frame depicts the bioluminescence signal intensity as a pseudocolor image superimposed on the gray-scale photographic image. The data are reported as total photon flux (p/s) from a circular region of interest (ROI). BLI signal intensity was monitored over 8 days after xenograft generation.

For the quantification of BLI data in the mouse xenograft model, values from according ROIs measured with CLI were subtracted from the raw BLI ROI values to obtain specific BLI signal intensities.

### Statistical Analysis

Data are presented as mean ± standard error of the mean (SEM). For the uptake experiments using the unicistronic, bicistronic and multicistronic vector after puromycin selection, two-way analysis of variance (ANOVA) statistical tests were performed with Bonferroni post-hoc tests. P-values<0.05 were considered statistically significant. BLI and CLI data were tested using an unpaired two-sided t-test after log transformation. Differentiation analysis was performed using a one-way ANOVA with Tukey post-hoc tests. The *in vivo* BLI data after intravenous injections were tested for gaussion distribution using the Kolmogorov-Smirnov test and the Pearson's R coefficient was calculated as a measure of linear correlation. The *in vivo* xenograft BLI data were evaluated using an unpaired t-test.

Data were processed using GraphPad Prism version 5.00 for Windows (GraphPad Software, San Diego, California, USA).

## Results

### hNIS is an efficient PET reporter in cell culture

**Figure 2:**
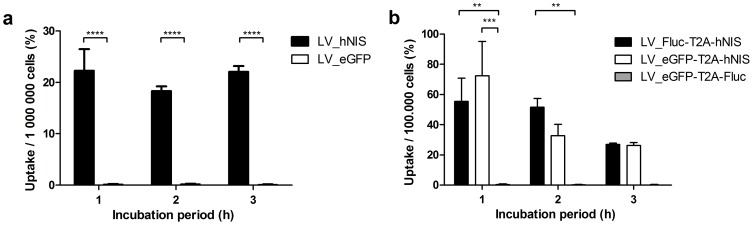
Results of uptake experiments with ^99m^TcO_4_^−^ in HEK293T cells after the induction of hNIS expression. Uptake ratios resulting from the transduction with a unicistronic LV (b) and a bicistronic LV (c). Significantly higher uptakes were observed in cells expressing hNIS. **: p<0.01; ***: p<0.001; ****:p<0.0001.

Different LV vector types (Table 1, Fig. 1) were tested in HEK293T cells for the optimization of the imaging reporter gene expression. Stably hNIS expressing cells or control cells were incubated with ^99m^TcO_4_^−^. All transfections and transductions (unicistronic LV and bicistronic LV, Fig. 2a,b) resulted in a 100-fold to 300-fold higher uptake of tracer in cells expressing the hNIS gene, compared to the controls (unicistronic LV: p<0.001; bicistronic LV: p<0.0001).

### EF1α as most potent promoter in MSC

**Figure 3:**
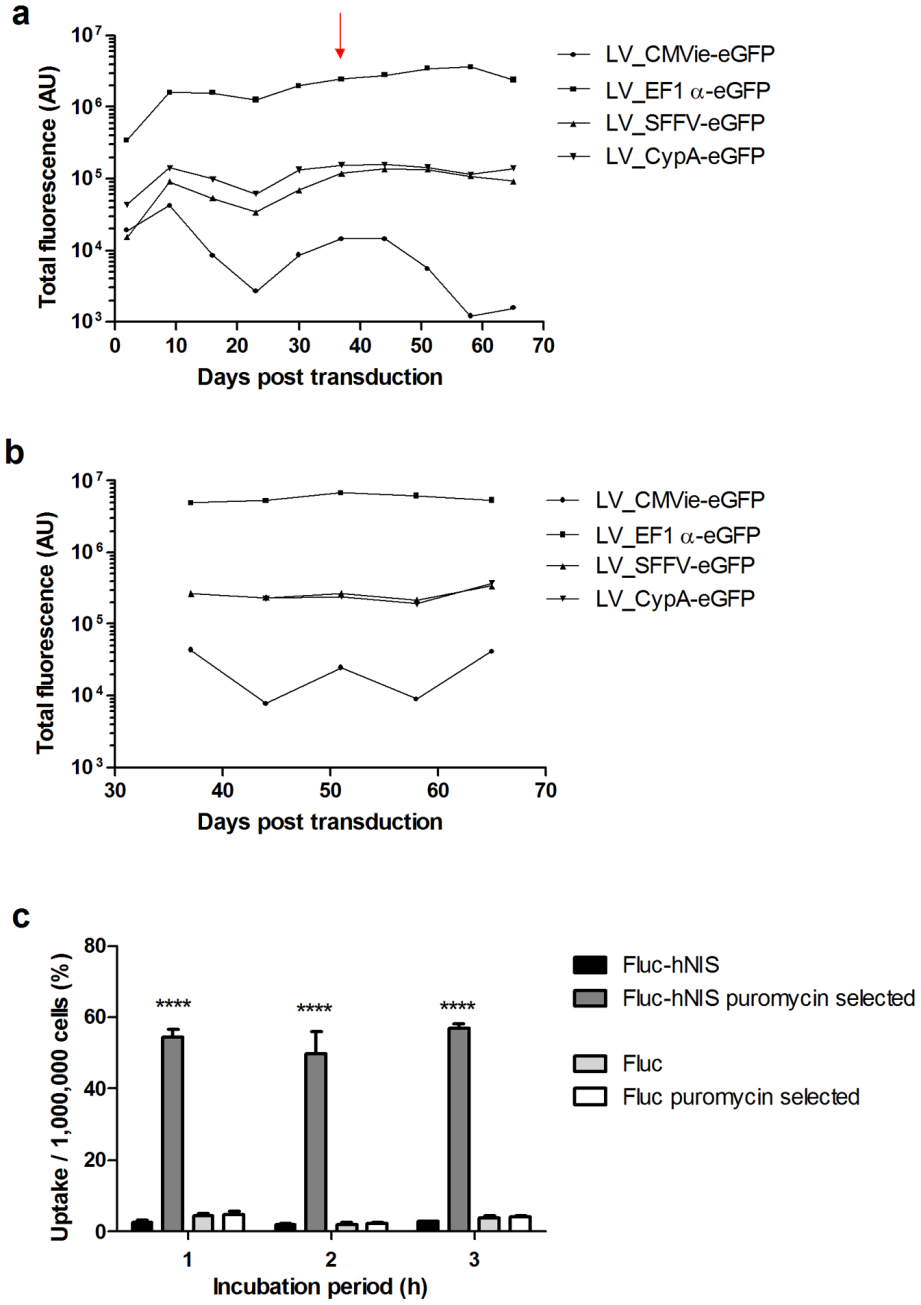
Optimization of LV constructs and selection. Different promoters were tested with FACS (AU: arbitrary units) for eGFP expression in MSCs transduced with different LV driven by different human or viral promoters (a). On day 38, FACS selection of the highest expressors was done (red arrow on panel (a)), and this population was also monitored (b). ^99m^TcO_4_^−^ uptake experiment in Fluc-hNIS or Fluc expressing MSCs with or without puromycin selection (c). ****:p<0.0001.

The optimal promoter allowing the highest expression in MSCs was assessed by comparing transduction of MSCs with LVs encoding eGFP driven by human promoters (EF1α and CypA), and viral promoters (CMVie, SFFV) (Fig. 3a,b). eGFP fluorescence was monitored with FACS, and there was a significantly higher expression level in cells transduced with the EF1α promoter, both without (Fig. 3a) and with sorting (Fig. 3b) of the 5 percent highest expressing cells (p<0.0001).

### Puromycin selection increases reporter gene expression

Cells transduced with the multicistronic LV constructs using the EF1α promoter were either kept on normal growth medium or on growth medium with puromycin. To determine the effect of puromycin selection of the expression of the imaging reporter genes, the uptake of ^99m^TcO_4_^−^ was assessed in both selected and non-selected cells (Fig. 3c). A significantly higher uptake of the radioligand was observed in Fluc-hNIS expressing MSCs upon selection with puromycin (p<0.0001), compared to nonselected and Fluc expressing MSCs.

### Validation of hNIS expression in MSCs

**Figure 4:**
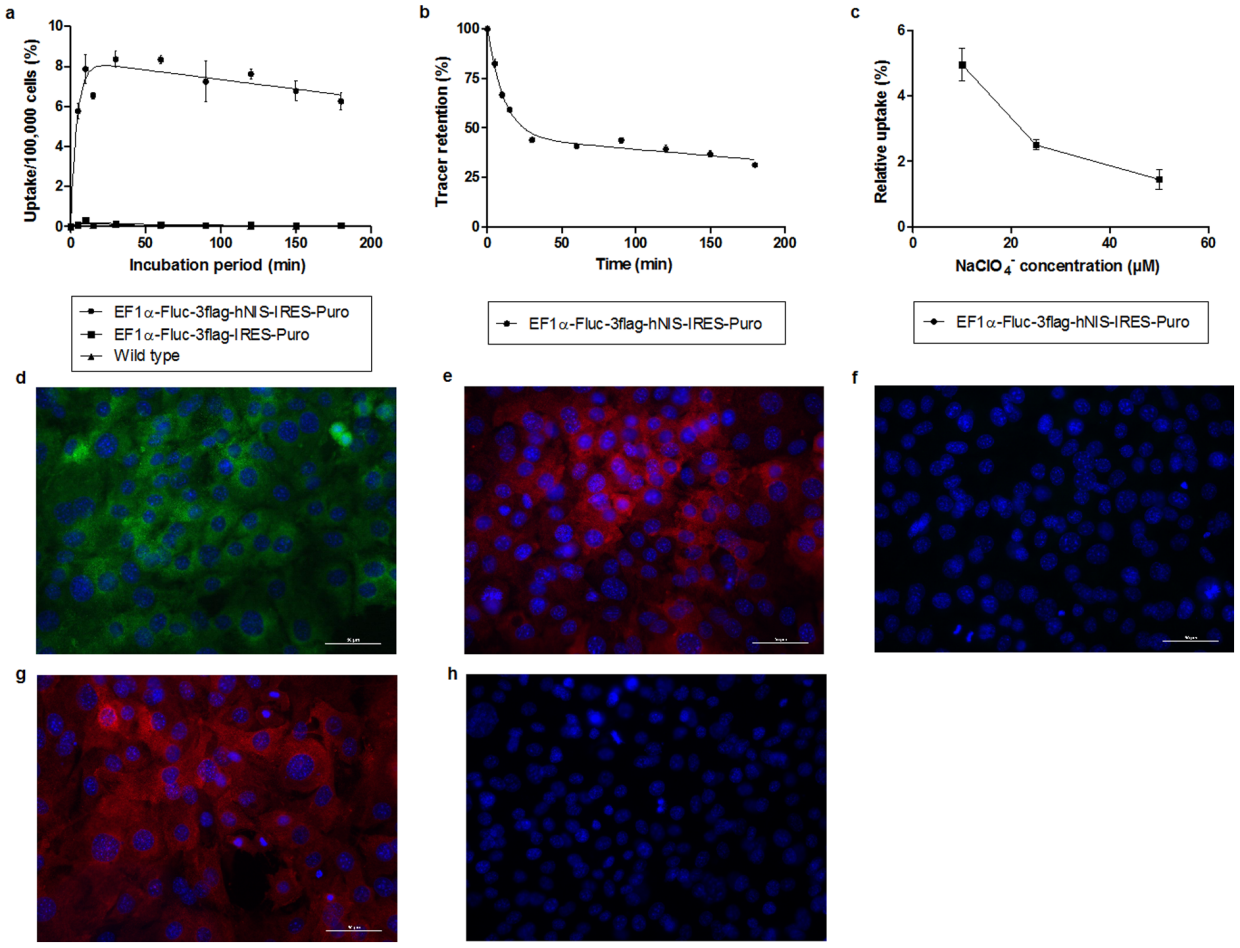
Validation of the multicistronic LV contruct. ^99m^TcO_4_^−^ uptake kinetics in MSCs transduced with the two different multicistronic LV constructs or wild type MSCs (a). Tracer elution from Fluc-hNIS expressing MSCs (b). The effect of different concentrations of NaClO_4_ on the uptake of ^99m^TcO_4_^−^ in Fluc-hNIS expressing MSCs (c). Immunocytochemistry against hNIS on Fluc-hNIS expressing MSCs and DAPI (d), against 3flag in 3flagFluc-hNIS expressing MSCs and DAPI (e) and negative control (f). Immunocytochemistry against 3flag in 3flagFluc expressing MSCs and DAPI (g) and negative control (h).

MSCs were transduced with the multicistronic LV, and the uptake kinetics of ^99m^TcO_4_^−^ were assessed. Untransduced MSCs and Fluc expressing MSCs were included as a negative control. Decay-corrected uptake data (Fig. 4a) showed a strong increase in uptake within the first 30 minutes of incubation in Fluc-hNIS expressing MSCs, which was followed by a state of equilibrium between the concentration inside and outside of the cells. A significantly higher uptake (75 and 120 fold increase) was observed in Fluc-hNIS expressing MSCs compared to either Fluc expressing MSCs or wild type MSCs (p<0.001).

The elution of the tracer from the cells after initial uptake was assessed in Fluc-hNIS expressing MSCs (Fig. 4b). After labeling, cells were incubated in tracer-free medium and the relative elution was calculated. A two-phased elution was observed, with a substantial tracer elution occurring within 30 minutes, followed by a slow elution of the tracer from the cells (p<0.001). After 3 hours, 31%±1.3% of the initially bound tracer was still present within the cells.

To determine the specificity of the uptake, the hNIS inhibitor NaClO_4_ was used (Fig. 4c). Cells were incubated with the tracer solved in the blocking solution and the uptake of ^99m^TcO_4_^−^ was measured. A significant decrease in the uptake of the tracer could be observed (p<0.01) after the administration of the blocker. Furthermore, the uptake of ^99m^TcO_4_^−^ decreased with increasing concentrations of NaClO_4_ (p<0.01). A decrease of 95%, 97.5% and 98.6% was observed with concentrations of 10, 20 and 50 µM of NaClO_4_, respectively. Data are represented as relative values compared to incubation without perchlorate.

The expression of the imaging reporter genes was confirmed by fluorescence immunocytochemistry. Fluc-hNIS expressing MSCs and Fluc expressing MSCs were stained with primary antibodies against hNIS and 3flag, and the expression of both reporter genes was observed (Fig. 4d,e,g). Negative controls did not show an aspecific staining or background signal (Fig. 4f,h).

### *In vitro* BLI and CLI

**Figure 5:**
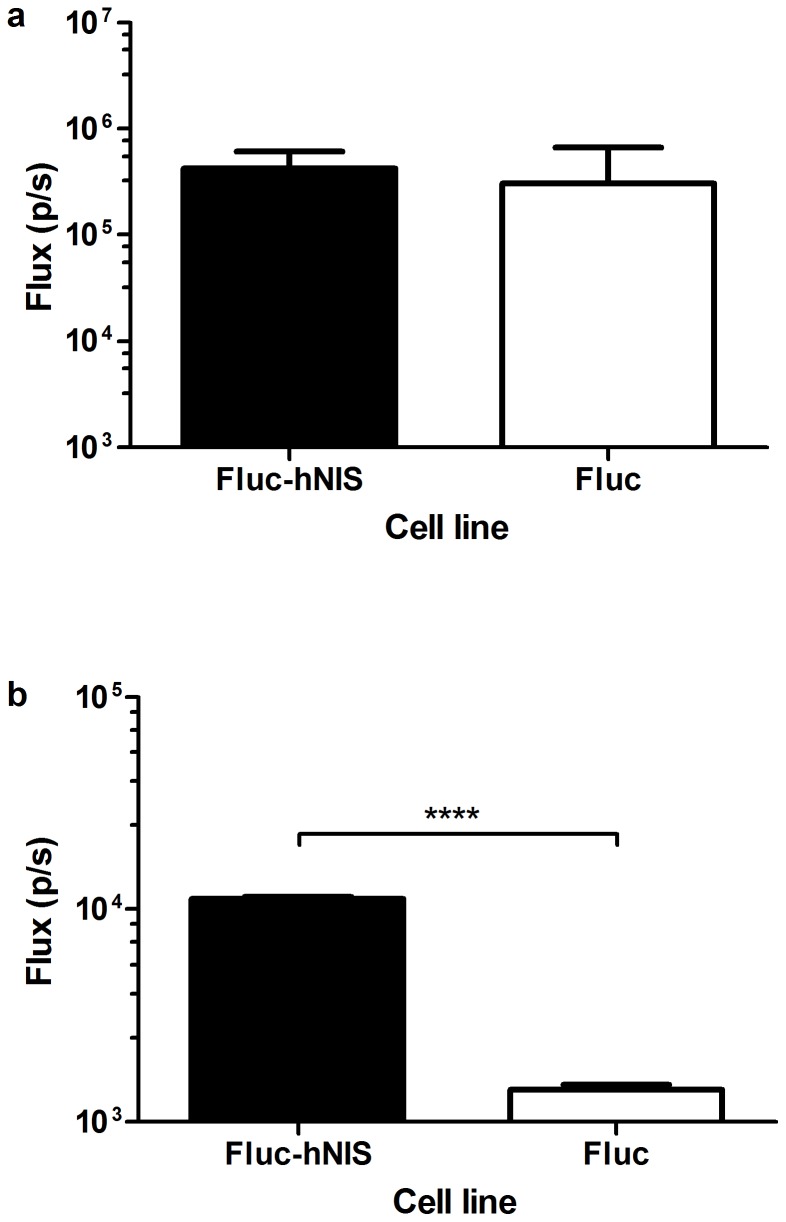
In vitro optical validation of the imaging reporter genes. In vitro BLI (a) and CLI (b) of Fluc-hNIS expressing MSCs and Fluc expressing MSCs. ****: p<0.0001.

*In vitro* BLI was performed to determine the expression of Fluc in the cells after transduction with both the Fluc-hNIS LV and the Fluc LV (Fig. 5a). Fluxes (in p/s) of log 5.63±0.42 and 5.49±0.9 were measured for the Fluc-hNIS and Fluc expressing cells, respectively. Hence, there was no significant difference in Fluc expression between both cell lines.

Both cell lines were also incubated with I^124^ and *in vitro* CLI was performed (Fig. 5b). A significant difference was observed between the fluxes of both cell lines (p<0.0001), with noted log values of 4.05±0.02 for the Fluc-hNIS expressing cells, and 3.15±0.06 for the Fluc expressing cells.

### MSC differentiations

**Figure 6:**
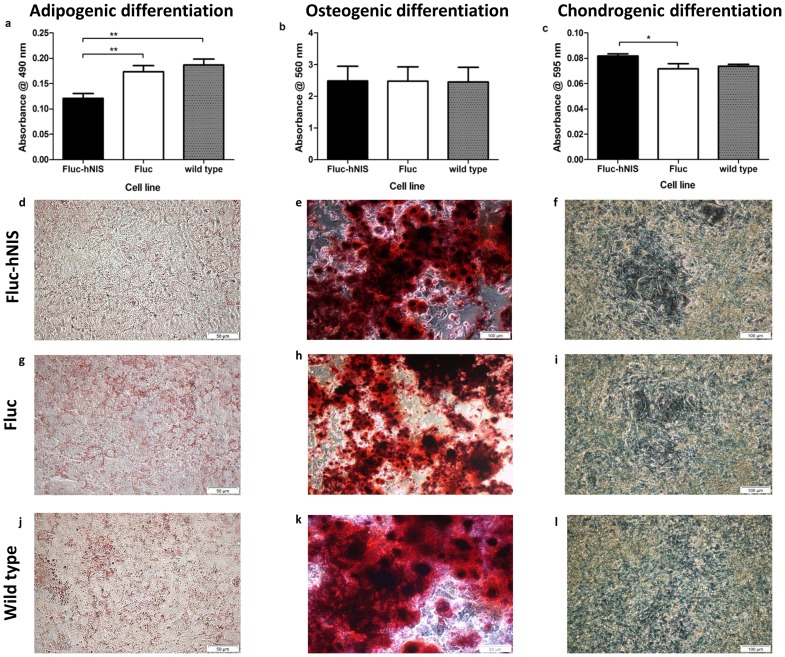
Adipogenic, osteogenic and chondrogenic differentiation capacity of Fluc-hNIS expressing MSCs, Fluc expressing MSCs and wild types. Extraction of the Oil red O dye following adipogenic differentiation measured at 490(a). Extraction of the Alizarin red S dye from differentiated osteoblast matrix, measured at 560 nm (b). Extraction of Alcian Blue dye from differentiated chondrocyte matrix, measured at 595 nm (c). *: p<0.05, **: p<0.01. Images of the Oil red O stainings (d,g,j), the Alizarin red S stainings (e,h,k) and the Alcian Blue stainings (f,I,l) for all conditions: Fluc-hNIS, Fluc expressing cells and wild types.

Fluc-hNIS expressing MSCs, Fluc expressing MSCs and wild type MSCs were differentiated towards the adipogenic, chondrogenic and osteogenic lineage (Fig. 6).

Quantification of the extracted oil red O dye resulted in following absorbance values (measured at 490 nm): 0.12, 0.17 and 0.19 for the Fluc-hNIS expressing MSCs, Fluc expressing MSCs or wild type MSCs, respectively, resulting in a respective difference of 35.3% and 7.4% compared to wild type MSCs (Fig. 6a,d,g,j). A significant difference between cells transduced with the Fluc-hNIS expressing MSCs and the two other cell lines was observed (p<0.01).

Following osteogenic differentiation, quantification of matrix mineralization resulted in following values after measuring the absorption of the samples at 560 nm: 2.49, 2.48 and 2.45 for the Fluc-hNIS expressing MSCs, Fluc expressing MSCs or wild type MSCs, respectively (Fig. 6b,e,h,k). Hence, no statistical difference was obtained (p>0.05).

Alcian blue staining following chondrogenic differentiation was quantified by dye extraction and measuring the absorption at 595 nm. The following values were obtained: 0.082, 0.072 and 0.074 for Fluc-hNIS expressing MSCs, Fluc expressing MSCs or wild type MSCs, respectively (Fig. 6c,f,i,l). A statistically significant increase in alcian blue dye incorporation within the chondrogenic micromasses was observed in the Fluc-hNIS expressing MSCs (p<0.05).

### Imaging of intravenously injected MSCs using BLI

**Figure 7:**
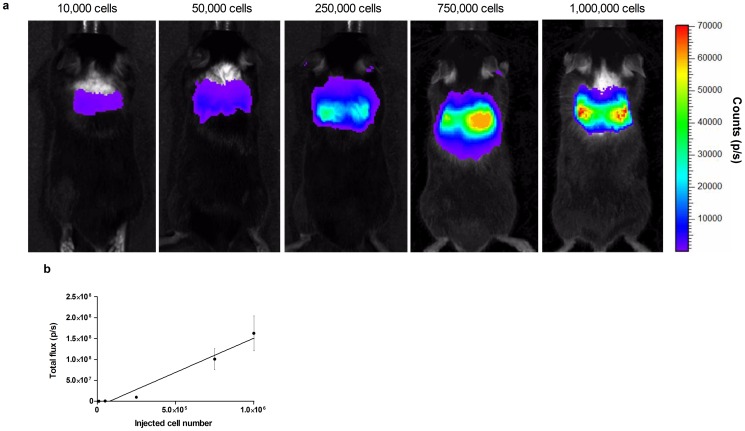
BLI data of intravenously injected Fluc-hNIS expressing MSCs. An accumulation of cells could be visualized in the lungs. Different cell numbers were injected, and total flux was measured. Both parameters were strongly correlated with an R^2^ of 0.97.

Different numbers of cells, ranging from 10,000 to 1,000,000, were injected in the tail vein of healthy C57BL/6 mice, and 30 minutes after cell injection, either BLI or small-animal PET was performed as a proof-of-principle for cell visualization. Using BLI, a robust signal could be obtained in the lungs. The signal intensity in the lungs increased when increasing cell amounts were injected intravenously. A significant correlation between injected amount of cells and total photon flux was obtained (p<0.01) with an R^2^ value of 0.97 (Fig. 7 a,b).

### Imaging of MSC xenografts using BLI, ^124^I small-animal PET and CLI

**Figure 8:**
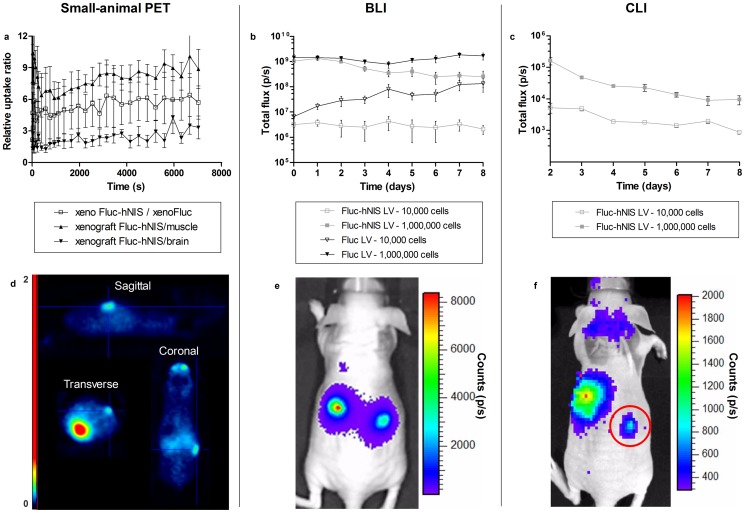
Visualization and follow-up of MSC xenografts. Fluc expressing MSCs were injected on the left flank of the body, and Fluc-hNIS expressing MSCs were engrafted on the right side of the body. Xenografts of 10,000 injected cells were injected near the front, and 1,000,000 cells were injected at the back for both conditions. Time activity curves of the ratios comparing the Fluc-hNIS expressing xenograft to background up tissues, showing higher expressions in the Fluc-hNIS expressing xenograft compared to background signals (a,d). BLI was also performed to follow the xenografts over time, with robust expression within all xenografts (b,e). CLI was performed to follow up the mice after ^124^I injection. The Fluc-hNIS expressing MSCs could be visualized and monitored over time (c,f).

MSCs expressing Fluc-hNIS or Fluc were engrafted subcutaneously in nude mice (n = 3), and imaged non-invasively using BLI, Cherenkov imaging and ^124^I small-animal PET (Fig. 8). On day 2 after cell injection, a ^124^I small-animal PET scan was performed to visualize the engrafted cells expressing Fluc-hNIS. A clear focus of increased tracer concentration was observed at the site where the 1,000,000 MSCs expressing Fluc-hNIS were injected. The xenograft resulting from the 10,000 cells could not be detected on the small-animal PET images (Fig. 8d).

The xenograft to background ratio was 8 when comparing the Fluc-hNIS expressing xenograft with muscle tissue. Five times more radiotracer uptake was seen compared to the control xenograft of 1,000,000 cells expressing Fluc. The brain showed 3 times less accumulation of the tracer compared to the Fluc-hNIS expressing xenograft (Fig. 8a).

The BLI signal in xenografts derived from 1,000,000 cells was significantly higher than in xenografts derived from 10,000 cells in both MSC conditions (p<0.001). However, robust and specific BLI signals could be obtained in all conditions over time, with no decrease in signal being perceived in the monitored time span of 8 days (Fig. 8b,e). The BLI signals coming from the Fluc-hNIS expressing MSCs and the Fluc expressing MSCs were within the same range.

Two days after xenograft injection, ^124^I small-animal PET was performed. In addition, ^124^I accumulation was monitored over time using CLI for 8 days. (Fig. 8c,f). Accumulation of ^124^I was detected in the thyroid, in the stomach and in the Fluc-hNIS expressing xenograft situated on the right side of the body (red circle). No signal was observed emanating from the bladder.

The Cherenkov signal detected in the xenograft expressing Fluc-hNIS with 1,000,000 cells persisted over the 8 days of measurements, decreasing according to the radioactive decay time of ^124^I (T1/2: 4.2 days) as was shown after an exponential fit with an R^2^ value of 0.63, yielding a half-life of 4.4 days.

## Discussion

The emergence of stem cell therapies in regenerative medicine urges the development of methodology for non-invasive tracking of engrafted cells. The ability to image MSCs would be beneficial as they hold clinical promise both due to their differentiation capacity as well as their possibility to exert immune modulation [5]–[7].

Stem cells expressing PET and SPECT imaging reporter genes can be visualized longitudinally *in vivo* using nuclear medicine imaging techniques. In this context, hNIS has been used already in several studies both in the context of diagnosis and therapy (for review [24]).

Tracking stem cells using imaging reporter genes requires the insertion of a functionally active transgene into the stem cells to obtain a functionally active reporter protein. LV are good candidates because the genes of interest are introduced into the genomic DNA of the host cells, and both dividing and non-dividing cells can be targeted [25].

For optimal expression of our reporter genes, we first optimized the transduction process of the MSCs as well as the promoter to drive the final construct. Here, the EF1α promoter resulted in the highest expression of eGFP, and was therefore chosen to drive our reporter construct. The optimization of a promoter for transductions has been shown to be of great importance. Other groups have also determined the optimal promoter for several purposes, and in different cell types the EF1α promoter proved to be a potent promoter with long-term expression on a high level outperforming other commonly used promoters [26]–[31].

The introduction of genes into the host cells' genetic material can result in adverse effects resulting in the disruption of biological processes. We evaluated the effect of the lentiviral transduction process on MSCs. Although some statistically significant differences were observed, the biological relevance is likely nihil. Therefore, we can conclude that lentiviral transduction with the presently used imaging reporter gene constructs does not hamper the differentiation capacity of the MSCs. Terrovitis et al have also shown that ectopic expression of hNIS using lentiviral vectors does not interfere with cell viability and function in cardiac-derived stem cells [32]. This was also shown by Hu et al, who showed that adenoviral vector transduction of the hNIS and rNIS gene had no adverse effects on MSCs [33].

We demonstrated that hNIS and Fluc expression is functional both *in vitro* and *in vivo*. *In vitro*, a robust radiotracer uptake of ^99m^TcO_4_^−^ was obtained with a steady state already at 30 minutes of incubation. The uptake was 75 or 120 times higher compared to control cells. Furthermore, NaClO_4_, an inhibitor of hNIS, was able to block the uptake of ^99m^TcO_4_^−^ in a dose-dependent manner.

However, a substantial elution from the cells was observed after labeling, with 31%±1.29% of the tracer being retained within the cells after 3 hours. This might be due to the initial strong concentration gradient that is generated while putting the cells containing the tracer on tracer-free medium, leading to a rapid washout of tracer molecules from the cells back into the medium, until the gradient between the cells and the new supernatant reaches a state of equilibrium. Furthermore, no organification of the ^99m^TcO_4_^−^ occurs after entry into the cells, as is the case in thyroidal tissue, and therefore no trapping of the molecules takes place. Nevertheless, 31% of the initially bound tracer molecules remained trapped within the cells after 3 hours.

As a proof-of-principle, Fluc-hNIS expressing MSCs were injected into the tail vein of healthy mice, and BLI signals in the lungs were measured. Due to the first pass mechanism, cells injected intravenously will first encounter the pulmonary capillary bed and will transiently reside in the lung (pre)capillary bed. The BLI signal correlated with the amount of cells injected, allowing non-invasive detection of the cells.

Finally, long-term noninvasive multimodality imaging was performed. For this purpose, 10,000 or 1,000,000 MSCs expressing Fluc or Fluc-hNIS were injected subcutaneously on the left flank and the right flank, respectively. All xenografts could be visualized using BLI, and monitored over time with a robust signal in all conditions. On day 2 after engraftment of the MSCs, ^124^I small-animal PET was performed, and the xenograft of 1,000,000 Fluc-hNIS expressing MSCs was clearly visible. The injection site of 10,000 cells was not visible because of the partial volume effect and the relatively higher background activity resulting from hNIS expressing organs and circulating tracer.

The accumulation of ^124^I was also monitored using CLI. These scans confirmed the information that was obtained with small-animal PET, because the cerenkov signal detected in the xenograft expressing Fluc-hNIS after injection of 1,000,000 cells persisted over the 8 days of measurement, decreasing according to the radioactive decay time of ^124^I (T1/2: 4.2 days) as shown by the exponential fit with an R^2^ value of 0.63, yielding a half-life of 4.4 days. From these data we can conclude that the ^124^I is trapped within the cells, and can be visualized using CLI. Besides the xenograft, also the stomach and the thyroid accumulated ^124^I, due to the endogenous hNIS expression in these tissues [1].

From these data we can conclude that imaging reporter genes are a suitable tool for the non-invasive visualization of stem cells after *in vivo* administration. Combining multiple imaging reporter genes in the same construct enables us to perform multimodality imaging to confirm the obtained data from one single imaging modality. hNIS was used as an imaging reporter gene together with Fluc. Using Fluc, longitudinal BLI can be performed as a traditional optical imaging modality. BLI features a very high sensitivity, almost no background signal, short scanning time, and thus high throughput imaging.

hNIS is a human reporter gene reducing the chances of immune responses against the reporter gene product. Furthermore, the expression of hNIS is restricted to a limited number of tissues, implying a relatively low background signal. hNIS can be used for both therapeutic and diagnostic purposes. Imaging can be performed with tracers for gamma cameras (^99m^TcO_4_^−^), which is widely available in every nuclear medicine department worldwide. The physical advantages of a PET camera can also be used as we have shown by using ^124^I. Furthermore, the gene can be used for therapeutic applications through the use of ^131^I.

When using multimodality imaging, it is also necessary to consider both the advantages and the disadvantages of each imaging modality. Optical imaging devices can be used for several purposes such as fluorescence, bioluminescence and more recently Cerenkov radiation. Here, CLI and BLI were used for cell tracking, and higher signal intensity could clearly be seen in BLI compared to CLI. Furthermore, BLI results in a very high signal to noise ratio due to the low background signals. In contrast, CLI images are noisy but CLI is a more translational optical imaging system than BLI, because it is using tracer molecules that are often already used in a clinical setting. This is particularly the case in preclinical imaging, as not every institute is equipped with costly dedicated small-animal nuclear imaging instruments.

For the imaging of radioactive tracer molecules, both CLI and small-animal PET can be used. The optical imaging equipment used for CLI allows a quick acquisition of data, and devices are more available compared to small-animal PET devices due to the lower costs of the hardware. However, PET imaging can also be performed in a relatively quick way, and results in the generation of tomographic images that are quantitatively more reliable. Furthermore, despite the efforts that have been taken for 3D optical imaging, there is a major lack of anatomical and tomographic data. Also, PET does not generate anatomical information, but this can be overcome relatively easily by co-registration of PET images to anatomical computed tomography (CT) images or magnetic resonance imaging (MRI) data. Indeed, the development of hybrid systems combining PET and either CT or MRI is an effective answer to this matter. Above all, PET is the only modality that can be used in patients, and therefore has the highest translational capacity of all the modalities.

For cell tracking studies, the combination of CLI and nuclear imaging might form a translational bridge between optical imaging and nuclear imaging modalities. Hence, the advantages of both techniques can be combined: quantitative information in a tomographic manner, and the high throughput and sensitivity of the optical imaging. We here show that hNIS is a suitable reporter gene for molecular imaging with PET and CLI. This reporter gene can be used in humans and is therefore a good candidate in translational studies.

Hence, future studies will include further reproducibility tests and the application of this system in disease models.

## References

[1] Dohan O, De la Vieja A, Paroder V, Riedel C, Artani M, et al (2003) The sodium/iodide Symporter (NIS): characterization, regulation, and medical significance. Endocr Rev 24: 48–77. 12588808 10.1210/er.2001-0029

[2] Van Sande J, Massart C, Beauwens R, Schoutens A, Costagliola S, et al (2003) Anion selectivity by the sodium iodide symporter. Endocrinology 144: 247–252. 12488351 10.1210/en.2002-220744

[3] Massoud TF, Gambhir SS (2003) Molecular imaging in living subjects: seeing fundamental biological processes in a new light. Genes Dev 17: 545–580. 12629038 10.1101/gad.1047403

[4] Ahn BC (2012) Sodium iodide symporter for nuclear molecular imaging and gene therapy: from bedside to bench and back. Theranostics 2: 392–402. 22539935 10.7150/thno.3722 PMC3337731

[5] Caplan AI (1991) Mesenchymal stem cells. J Orthop Res 9: 641–650. 1870029 10.1002/jor.1100090504

[6] Pittenger MF, Mackay AM, Beck SC, Jaiswal RK, Douglas R, et al (1999) Multilineage potential of adult human mesenchymal stem cells. Science 284: 143–147. 10102814 10.1126/science.284.5411.143

[7] Barry FP, Murphy JM (2004) Mesenchymal stem cells: clinical applications and biological characterization. Int J Biochem Cell Biol 36: 568–584. 15010324 10.1016/j.biocel.2003.11.001

[8] Uccelli A, Pistoia V, Moretta L (2007) Mesenchymal stem cells: a new strategy for immunosuppression? Trends Immunol 28: 219–226. 17400510 10.1016/j.it.2007.03.001

[9] Caplan AI (2007) Adult mesenchymal stem cells for tissue engineering versus regenerative medicine. J Cell Physiol 213: 341–347. 17620285 10.1002/jcp.21200

[10] Dwyer RM, Khan S, Barry FP, O'Brien T, Kerin MJ (2010) Advances in mesenchymal stem cell-mediated gene therapy for cancer. Stem Cell Res Ther 1: 25. 20699014 10.1186/scrt25 PMC2941117

[11] Kumar S, Chanda D, Ponnazhagan S (2008) Therapeutic potential of genetically modified mesenchymal stem cells. Gene Ther 15: 711–715. 18356815 10.1038/gt.2008.35

[12] Nakamizo A, Marini F, Amano T, Khan A, Studeny M, et al (2005) Human bone marrow-derived mesenchymal stem cells in the treatment of gliomas. Cancer Res 65: 3307–3318. 15833864 10.1158/0008-5472.CAN-04-1874

[13] Hare JM, Traverse JH, Henry TD, Dib N, Strumpf RK, et al (2009) A randomized, double-blind, placebo-controlled, dose-escalation study of intravenous adult human mesenchymal stem cells (prochymal) after acute myocardial infarction. J Am Coll Cardiol 54: 2277–2286. 19958962 10.1016/j.jacc.2009.06.055 PMC3580848

[14] Bernardo ME, Ball LM, Cometa AM, Roelofs H, Zecca M, et al (2011) Co-infusion of ex vivo-expanded, parental MSCs prevents life-threatening acute GVHD, but does not reduce the risk of graft failure in pediatric patients undergoing allogeneic umbilical cord blood transplantation. Bone Marrow Transplant 46: 200–207. 20400983 10.1038/bmt.2010.87

[15] Kebriaei P, Isola L, Bahceci E, Holland K, Rowley S, et al (2009) Adult human mesenchymal stem cells added to corticosteroid therapy for the treatment of acute graft-versus-host disease. Biol Blood Marrow Transplant 15: 804–811. 19539211 10.1016/j.bbmt.2008.03.012

[16] Le Blanc K, Frassoni F, Ball L, Locatelli F, Roelofs H, et al (2008) Mesenchymal stem cells for treatment of steroid-resistant, severe, acute graft-versus-host disease: a phase II study. Lancet 371: 1579–1586. 18468541 10.1016/S0140-6736(08)60690-X

[17] Reinders ME, de Fijter JW, Roelofs H, Bajema IM, de Vries DK, et al (2013) Autologous bone marrow-derived mesenchymal stromal cells for the treatment of allograft rejection after renal transplantation: results of a phase I study. Stem Cells Transl Med 2: 107–111. 23349326 10.5966/sctm.2012-0114 PMC3659754

[18] Ringden O, Uzunel M, Rasmusson I, Remberger M, Sundberg B, et al (2006) Mesenchymal stem cells for treatment of therapy-resistant graft-versus-host disease. Transplantation 81: 1390–1397. 16732175 10.1097/01.tp.0000214462.63943.14

[19] Thorek D, Robertson R, Bacchus WA, Hahn J, Rothberg J, et al (2012) Cerenkov imaging - a new modality for molecular imaging. Am J Nucl Med Mol Imaging 2: 163–173. 23133811 PMC3477724

[20] Xu Y, Liu H, Cheng Z (2009) Harnessing the power of radionuclides for optical imaging: Cerenkov luminescence imaging. J Nucl Med 52: 2009–2018. 10.2967/jnumed.111.092965 22080446

[21] Peister A, Mellad JA, Larson BL, Hall BM, Gibson LF, et al (2004) Adult stem cells from bone marrow (MSCs) isolated from different strains of inbred mice vary in surface epitopes, rates of proliferation, and differentiation potential. Blood 103: 1662–1668. 14592819 10.1182/blood-2003-09-3070

[22] Ibrahimi A, Vande Velde G, Reumers V, Toelen J, Thiry I, et al (2009) Highly efficient multicistronic lentiviral vectors with peptide 2A sequences. Hum Gene Ther 20: 845–860. 19419274 10.1089/hum.2008.188

[23] Geraerts M, Willems S, Baekelandt V, Debyser Z, Gijsbers R (2006) Comparison of lentiviral vector titration methods. BMC Biotechnol 6: 34. 16836756 10.1186/1472-6750-6-34 PMC1534021

[24] Cho JY (2002) A transporter gene (sodium iodide symporter) for dual purposes in gene therapy: imaging and therapy. Curr Gene Ther 2: 393–402. 12477251 10.2174/1566523023347599

[25] Sakuma T, Barry MA, Ikeda Y (2012) Lentiviral vectors: basic to translational. Biochem J 443: 603–618. 22507128 10.1042/BJ20120146

[26] Dupuy FP, Mouly E, Mesel-Lemoine M, Morel C, Abriol J, et al (2005) Lentiviral transduction of human hematopoietic cells by HIV-1- and SIV-based vectors containing a bicistronic cassette driven by various internal promoters. J Gene Med 7: 1158–1171. 15880619 10.1002/jgm.769

[27] Kim S, Kim GJ, Miyoshi H, Moon SH, Ahn SE, et al (2007) Efficiency of the elongation factor-1alpha promoter in mammalian embryonic stem cells using lentiviral gene delivery systems. Stem Cells Dev 16: 537–545. 17784828 10.1089/scd.2006.0088

[28] Petkov S, Hyttel P, Niemann H (2013) The choice of expression vector promoter is an important factor in the reprogramming of porcine fibroblasts into induced pluripotent cells. Cell Reprogram 15: 1–8. 23379578 10.1089/cell.2012.0053

[29] Ramezani A, Hawley TS, Hawley RG (2000) Lentiviral vectors for enhanced gene expression in human hematopoietic cells. Mol Ther 2: 458–469. 11082319 10.1006/mthe.2000.0190

[30] Varma NR, Janic B, Ali MM, Iskander A, Arbab AS (2011) Lentiviral Based Gene Transduction and Promoter Studies in Human Hematopoietic Stem Cells (hHSCs). J Stem Cells Regen Med 7: 41–53. 21743782 10.46582/jsrm.0701005 PMC3130352

[31] Wang R, Liang J, Jiang H, Qin LJ, Yang HT (2008) Promoter-dependent EGFP expression during embryonic stem cell propagation and differentiation. Stem Cells Dev 17: 279–289. 18447643 10.1089/scd.2007.0084

[32] Terrovitis J, Kwok KF, Lautamaki R, Engles JM, Barth AS, et al (2008) Ectopic expression of the sodium-iodide symporter enables imaging of transplanted cardiac stem cells in vivo by single-photon emission computed tomography or positron emission tomography. J Am Coll Cardiol 52: 1652–1660. 18992656 10.1016/j.jacc.2008.06.051 PMC4980098

[33] Hu S, Cao W, Lan X, He Y, Lang J, et al (2011) Comparison of rNIS and hNIS as reporter genes for noninvasive imaging of bone mesenchymal stem cells transplanted into infarcted rat myocardium. Mol Imaging 10: 227–237. 21518634 10.2310/7290.2010.00051

